# MIGREW: database on molecular identification of genes for resistance in wheat

**DOI:** 10.1186/s12859-018-2569-4

**Published:** 2019-02-05

**Authors:** Fedor V. Kazantsev, Ekaterina S. Skolotneva, Vasiliy N. Kelbin, Elena A. Salina, Sergey A. Lashin

**Affiliations:** 1 Institute of Cytology and Genetics SB RAS, Novosibirsk, Russia; 2 Novosibirsk State University, Novosibirsk, Russia

**Keywords:** Wheat, Stem rust, Resistant genes, Avirulent genes, Database

## Abstract

Population structure of fungal infections in wheat differs between wheat varieties and environments. Taking into account evolution of host-pathogen interactions, genetic diversity of both wheat and fungus must be a monitored. In order to catalogue information to support need of wheat pathologists and breeders, who use conventional methods and Molecular Assisted Selection (MAS) techniques, we have developed the Molecular Identification of Genes for Resistance in Wheat (MIGREW) database. The main goal of this database is to support wheat breeding efforts to develop immunity to rusts and powdery mildew. MIGREW is also focused on effectiveness of wheat resistance genes in different regions of Russia to provide users relevant information on the rapidly changing population structure of pathogens.

**Electronic supplementary material:**

The online version of this article (10.1186/s12859-018-2569-4) contains supplementary material, which is available to authorized users.

## Background

Wheat is one of the leading crops in Russia and worldwide (http://www.fao.org/fileadmin/templates/est/meetings/wto_comm/Trade_Policy_Brief_Russia_final.pdf). In most agricultural regions of the Russian Federation, the wheat pathogen complex affecting the plant organs containing chlorophyll is represented by the following species: *Puccinia triticina* causing leaf rust; *P. graminis* causing stem rust; and *Blumeria graminis* causing powdery mildew. Population structure of fungal pathogens depends on environment and wheat genotype. Taking into account the evolution of host-pathogen interactions [1, 2], genetic diversity of wheat and fungus must be monitored. Plant disease resistance detect pathogen attacks and facilitate a counter strike against pathogens [3–6]. The information on avirulent genes (Avr) in migrating fungal populations together with the resistant genes (R) and translocations in wheat lines is important to be incorporated into breeding programs. However, this pool of data is hard to use without integrative system. There is a lack of available database for Russian wheat varieties and breeding lines genotyped for resistant genes and no open access for the information of virulence genes in pathogen populations spreading on wheat crops.

Regarding the diversity of Russian wheat germplasm, there is a unique database GRIS developed at the Vavilov Research Institute. GRIS (http://wheatpedigree.net) is a Genetic Resources Information System for Wheat and Triticale, which provides information services for breeding and research programs containing the data on more than 170,000 genotypes (varieties and breeding lines) from national germplasm collections and research laboratories. Having an undoubted value for utilization by wheat geneticists, this database, however, does not fully meet the interests of both breeders and plant pathologists who deal with wheat breeding programs for immunity.

Since the modern technology of Marker Assisted Selection (MAS) complemented conventional selection methods, there is an urgent need to make the MAS technique widely available. A brilliant example of such an information resource is an open database MASwheat, developed in the USA. The MASwheat (http://maswheat.ucdavis.edu/) provides an extensive list of protocols for more than 40 molecular markers for resistance genes in wheat. However, before applying in regional breeding program, all molecular markers loaded in the MASwheat should be verified on an extended array of the Russian germplasm.

In order to catalogue information serving the interests of Russian wheat pathologists and breeders, which use conventional methods and MAS techniques, we have developed the MIGREW (Molecular Identification of Genes for Resistance in Wheat) database. The main goal of the proposed database is to increase the potentials of national wheat breeding for immunity to rusts and powdery mildew. The MIGREW also focuses on effectiveness of wheat resistance genes in different regions of Russia to make the database more adequate to the rapidly changing population structure of pathogens.

### Database organization and content

**Fig. 1:**
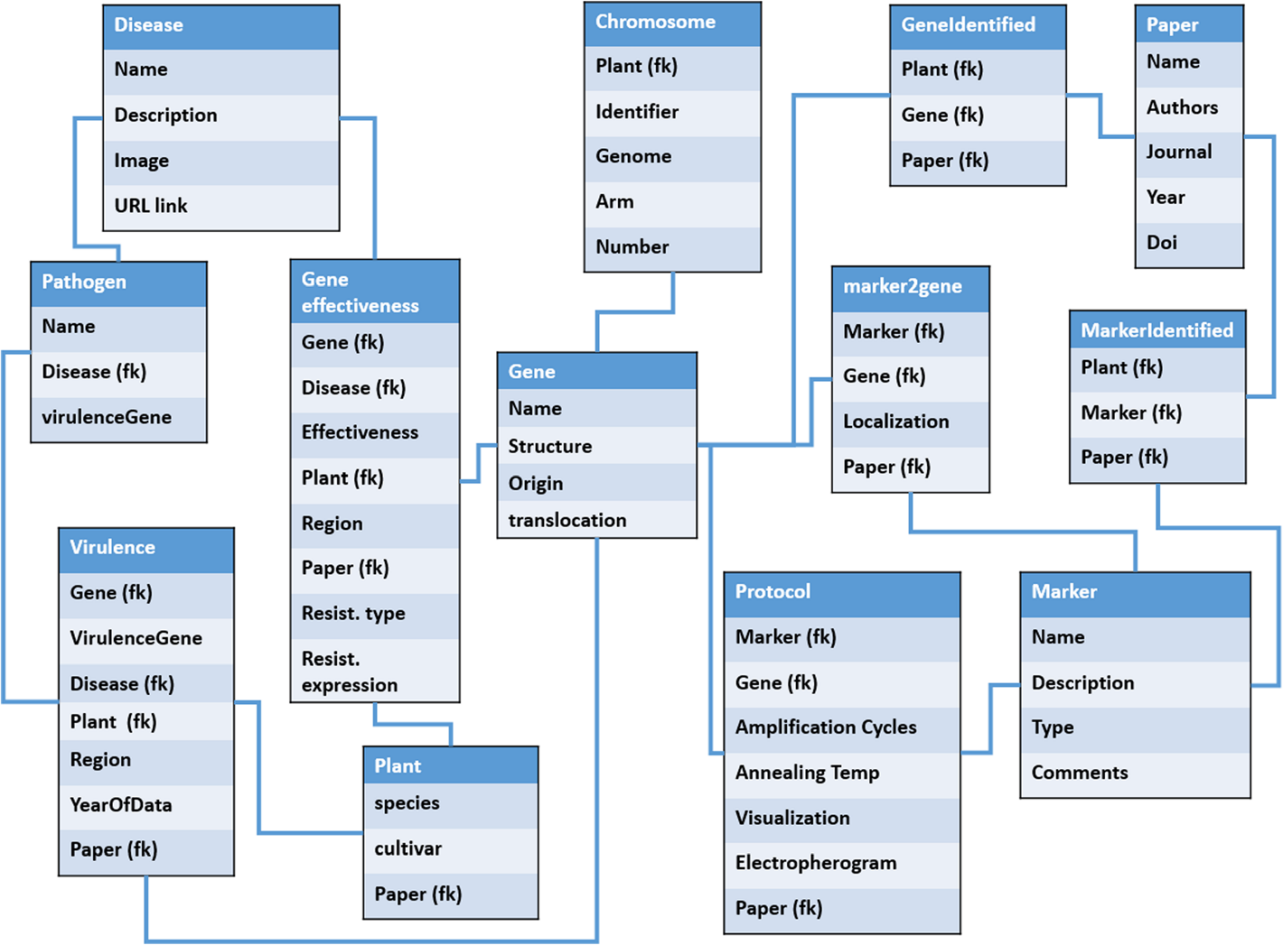
Simplified MIGREW database scheme. The table fields with “(fk)” mark points to connection with related database object

MIGREW is developed in classical MVC (Model-View-Controller) architecture. The model layer is the PostgreSQL database containing 16 tables. The View layer is a WEB application that designed in Java Script language with Webix (webix.com) libraries. The Controller layer is the java application designed using *spring.io* libraries that performs REST API access to the data. On a simplified database scheme (see Fig. 1) one can find data structures and main connections between objects.

In the database, we have the following objects: diseases, plants, chromosomes, genes, markers, protocols, pathogens and papers. We have built advanced relations between objects. It makes possible to get the information in the following manner: *For the Stem Rust disease we have X genes, that have own marks of resistance in selected regions. This phenomenon has been shown in the list of publications.* In addition, we can go deeper. For each object of the database, we can obtain all levels of related objects. Some typical use cases with the database we have implemented in the web application.

In addition, one can use the MIGREW REST API for direct data access with any programming/modeling tool that support REST service calls (Python, R or Matlab, for instance). The web application uses same API so one can build own tool for the MIGREW data exploring. The example of using the MIGREW REST API with Python can be found in Additional file 1.

The MIGREW was initially loaded with the published data [7–15] along with our original unpublished data. Most of the entities in MIGREW has connection with data sources. Many of that publications have no yet been digitized (books and articles that has not been scanned into the digital form as .pdf or other), and, perhaps, never will.

Nowadays the database contains information extracted from 50 papers for the period from 1973 to 2018. The data on 4 diseases and 3 genes associated with them. There are 45 pyramided genotypes (noted as gene pyramids in the database), that combined from at least one of those genes; 11 markers that found for these genes; and 502 plants varieties.

### Utility and discussion

**Fig. 2:**
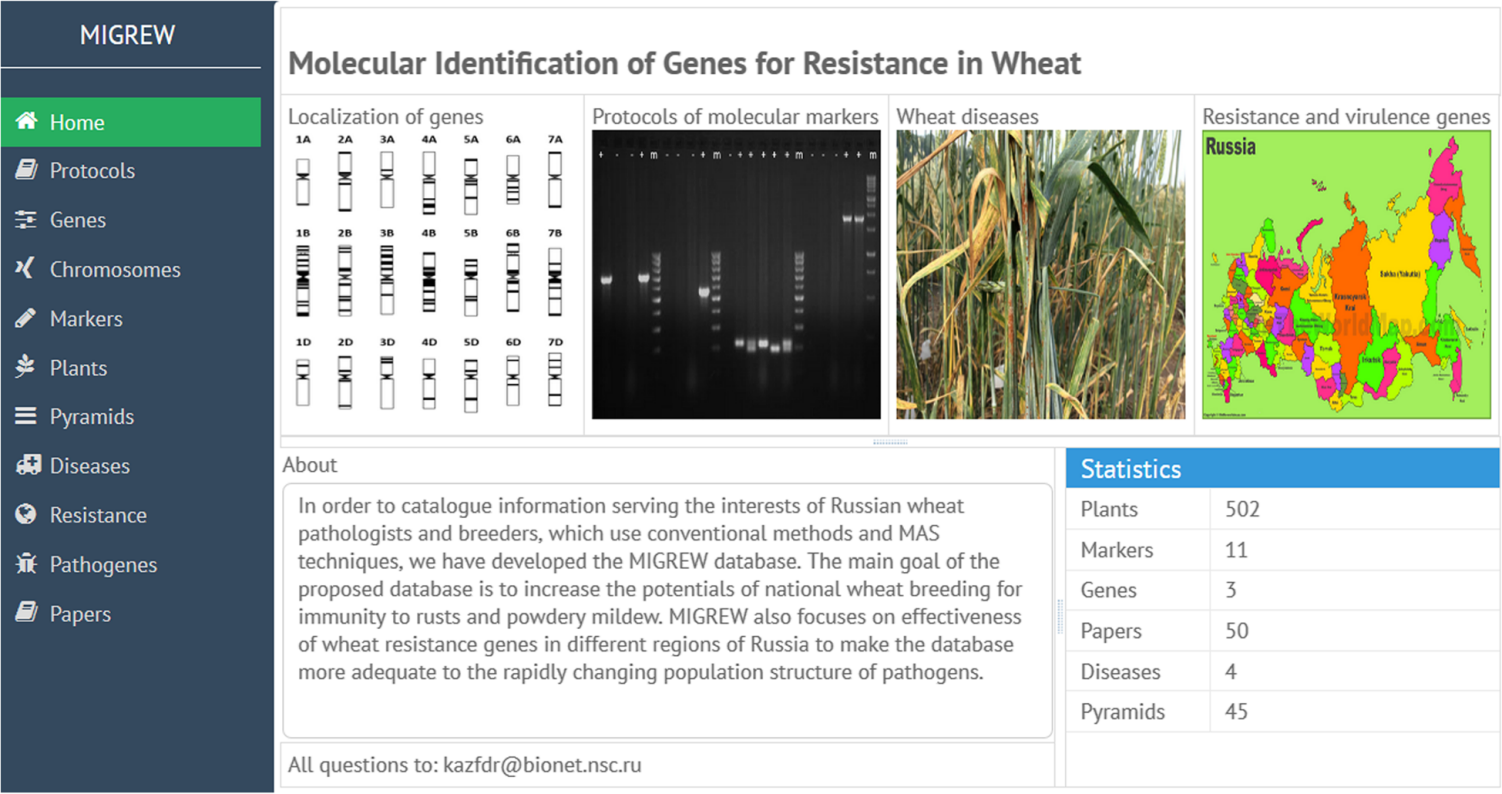
The MIGREW homepage

The MIGREW home page (*migrew.sysbio.cytogen.ru*) provides an easy access to any part of the database. It contains basic information as Database Statistics, About and Feedback pages (Fig. 2).

**Fig. 3:**
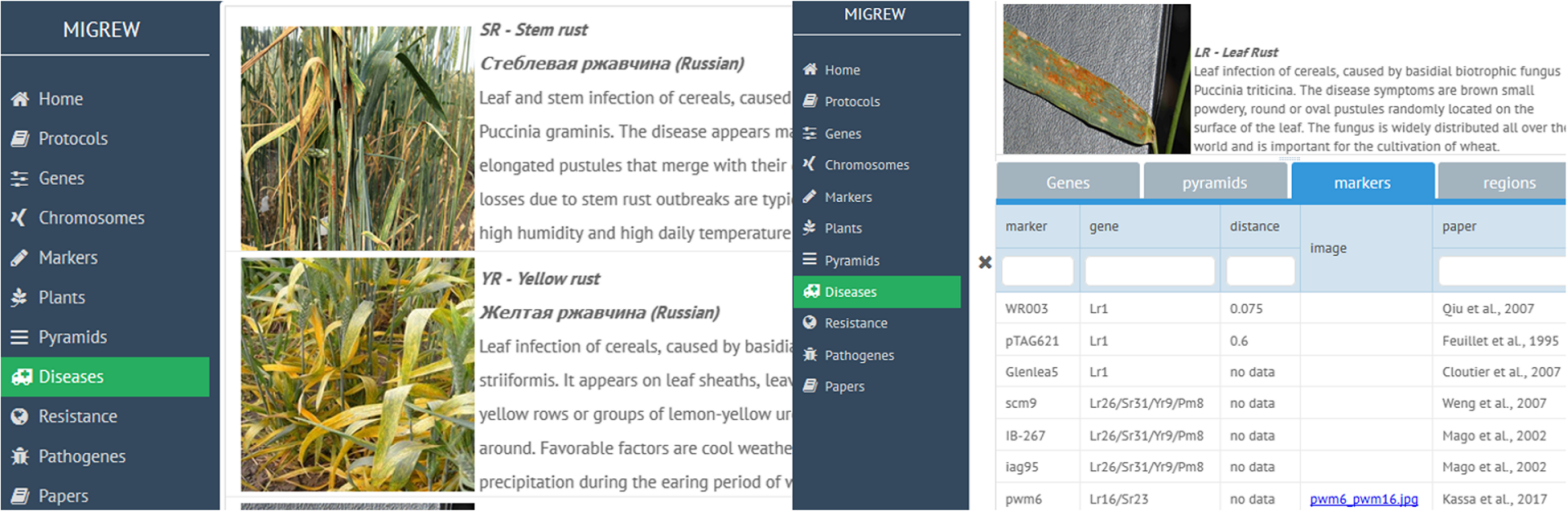
Diseases list page (left). Disease description page (right) with associated object tabs

On the homepage, the typical data exploring directions were placed: Localization of genes, Protocols of molecular markers, Wheat diseases, resistance and virulence genes. On the left side, there is a main navigation menu with data domains: “home, protocols, genes, chromosomes, markers, plants, pyramids, diseases, resistance, pathogens, and papers”. They are labeled separately to represent the stored information related to the specific tab. Each tab content has the similar structure of data visualization: the list of available domain objects, on the main page; and the single object description, that appears after double clicking on the list element. Moreover, the Description page contains the tabs with list of related objects. The “Diseases” domain (Fig. 3) is a good illustration of such an interface.

Selection of the “Diseases” option shows the list of fungal diseases of wheat stored in the database. By clicking the specific disease tab, one can get the diseases description page (Fig. 3, right) and obtain the following information: the list of wheat resistance genes and their effectiveness dependent on different regions of the Russian Federation, the wheat varieties and breeding lines carrying the resistance genes as single and as in pyramids with others; distribution of virulence in the pathogen population on the map of the Russian Federation.

**Fig. 4:**
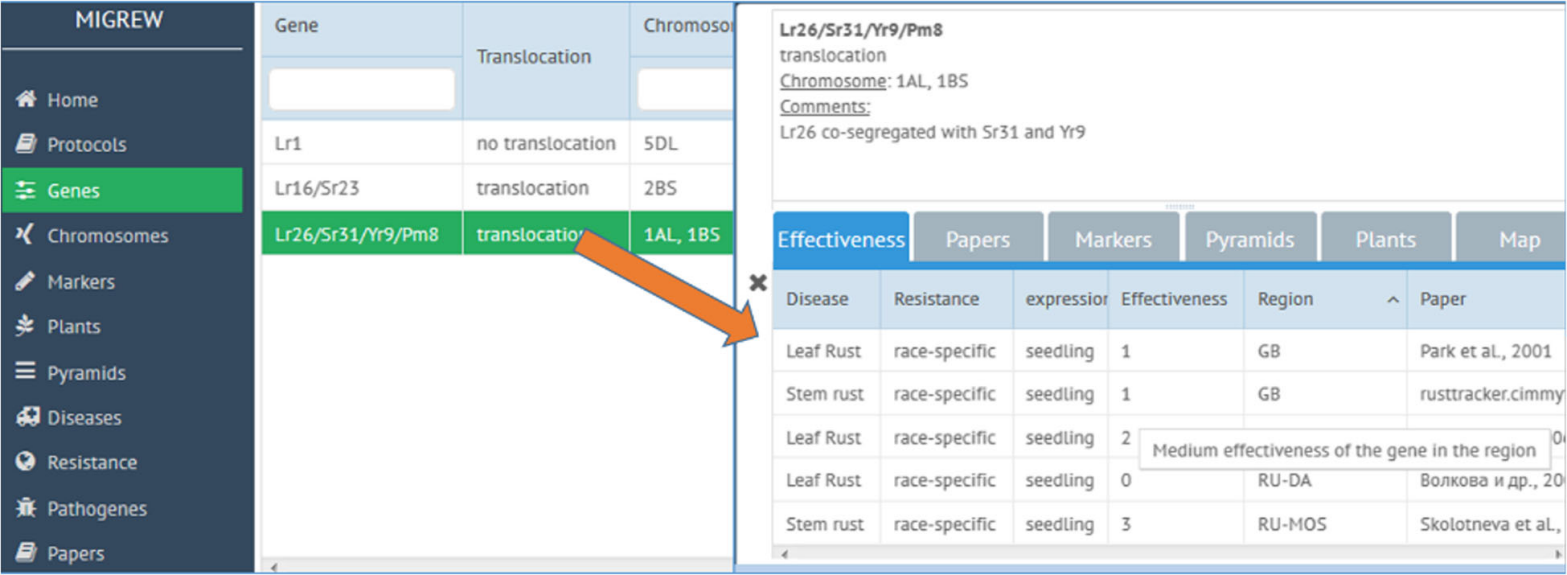
Genes list page (left). Selected gene description page (right) with associated object tabs

After choosing “Genes” tab in the menu (Fig. 4), one can see the list of resistance genes available in the database as well as other basic properties of these genes, such as translocation state and localization on the chromosome. Across the page, there is a search bar for gene symbols. By double-clicking on the tag of the interesting gene, one will open the page with additional information: publications where the gene is mentioned, effectiveness against diseases, estimated in 0–4 scale; molecular markers for identification the gene; pyramids containing this gene; plants where this gene was identified with links to the corresponding publications.

Another way to get a gene information is to request it via chromosome. It can be done by choosing “Chromosome” tab in the menu. Across the page, there is a search bar for chromosomes. The bar also supports filtering chromosomes by the name of genome (A, B, D), the number of chromosome (1–7) and chromosome arm (large or short). The selected chromosome description page contains list of genes, which are carried on the target fragment of wheat genome.

**Fig. 5:**
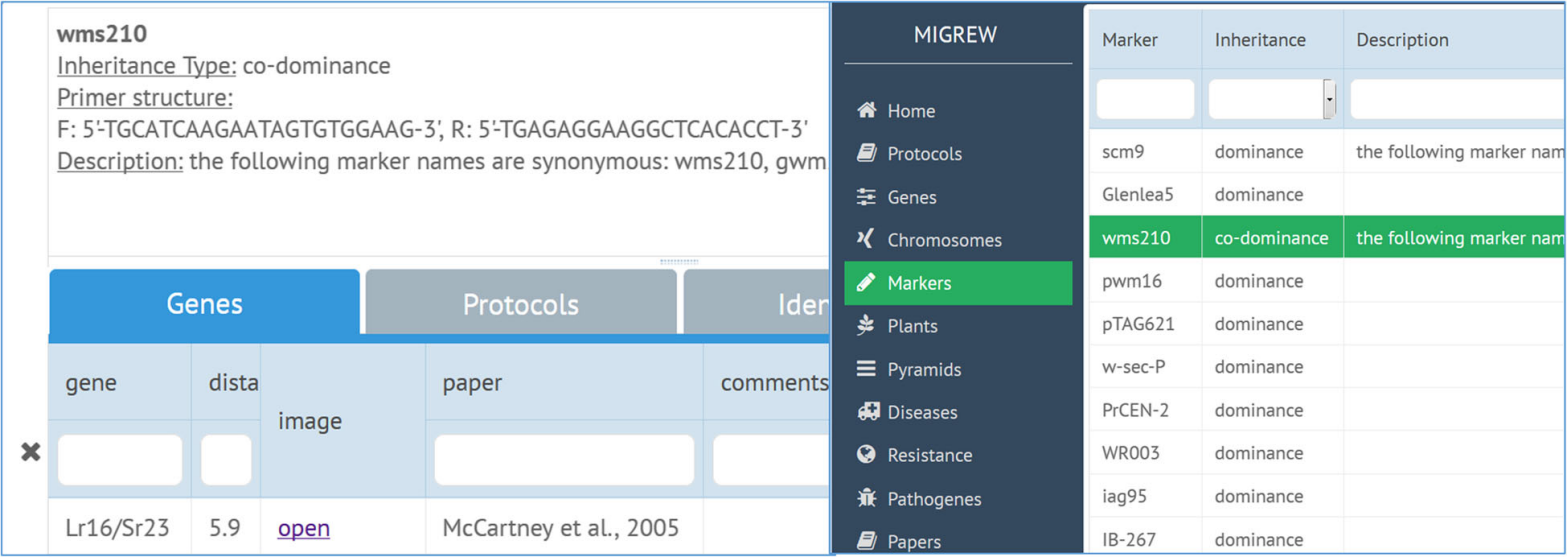
Markers list page (left). Marker description page (right) with associated object tabs

After choosing “Markers” tab in the menu, one can see the list of available markers stored in the database and their basic properties such as inheritance type, structure and a short description (Fig. 5, left). Across the page, there is a search bar for marker symbols. By double-clicking on the tag of the interesting marker, one opens the page with more information (Fig. 5, right): corresponded publications, genes that it marks; marker protocol; plants where this marker was identified. Markers, verified by the Laboratory of Plant Molecular Genetics and Cytogenetics at the Institute of cytology and genetics SB RAS, are illustrated with original electropherograms. There is an option in the MIGREW to contribute the data of markers, which verified in another laboratory.

**Fig. 6:**
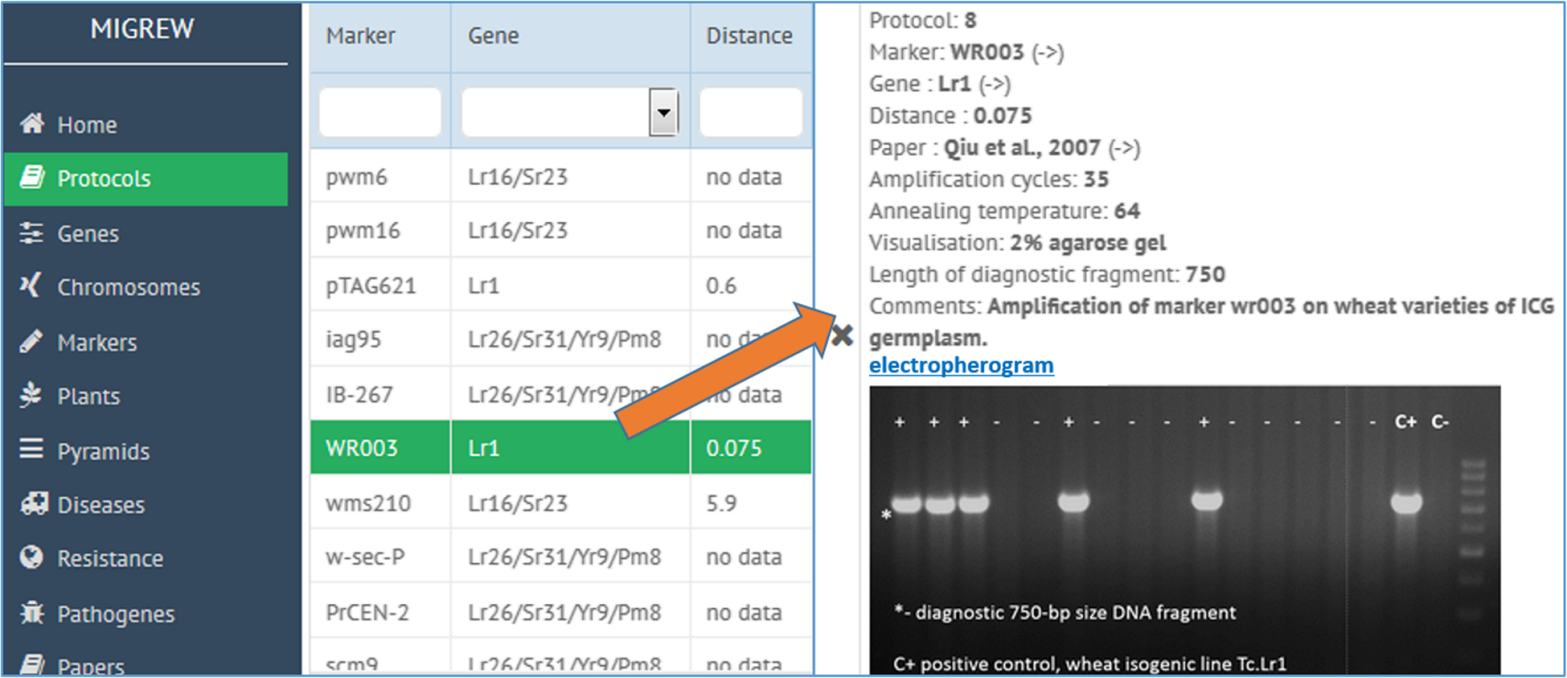
Protocols list page (left). Protocol description page (right)

After choosing “Protocols” tab in the menu (Fig. 6, left), one can find the list of molecular markers and corresponding resistance genes. Double-click on a marker or a gene tag shows the protocol data, which includes amplification cycles, annealing temperature, visualization system and length of PCR diagnostic fragment (Fig. 6, right).

**Fig. 7:**
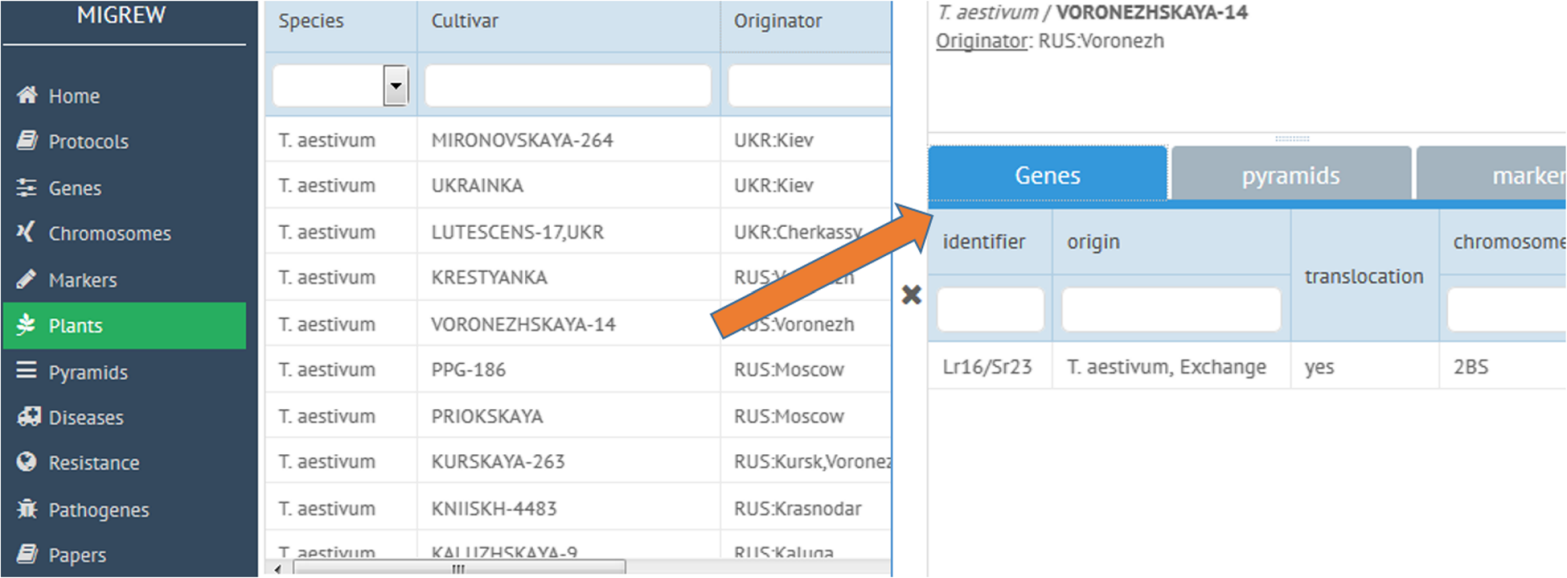
Plants list page (left). Plant description page (right) with associated object tabs

After choosing “Plants” tab in the menu (Fig. 7, left), one can see the list of plants stored in the database. Double-click on a certain plant opens the page with additional information (Fig. 7, right) such as: originator of variety genes and markers identified in this plant genotype, effectiveness against diseases and links to corresponded publications.

**Fig. 8:**
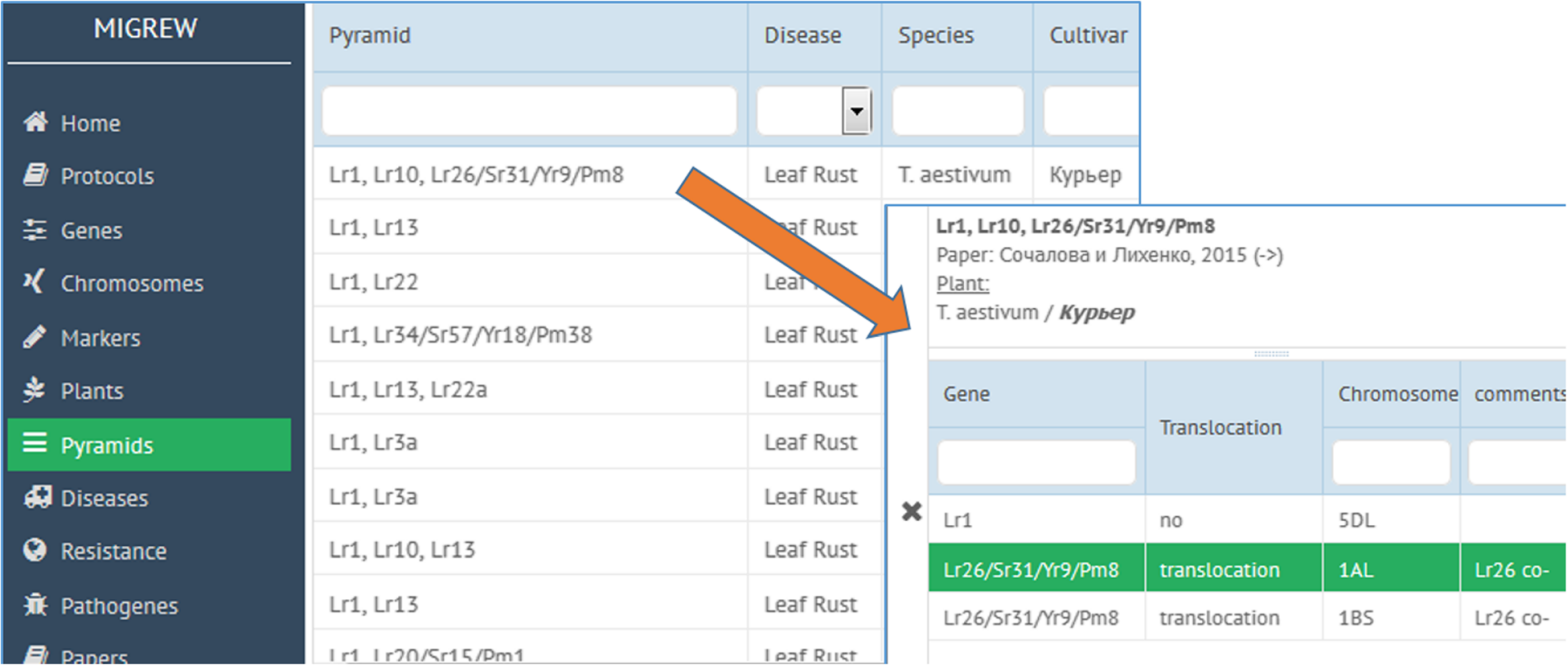
Pyramids list page (left). Pyramid description page (right) with associated genes

After choosing “Pyramids” tab in the menu (Fig. 8, left), one can see the list of gene pyramids represented in wheat varieties. On the top of the page, there is a search bar for gene symbols to search for a gene pyramid in which a gene under interest is involved (Fig. 8, right). Selected pyramid description page contains not only the corresponding paper and the plant, but also links to the genes forming this pyramid.

**Fig. 9:**
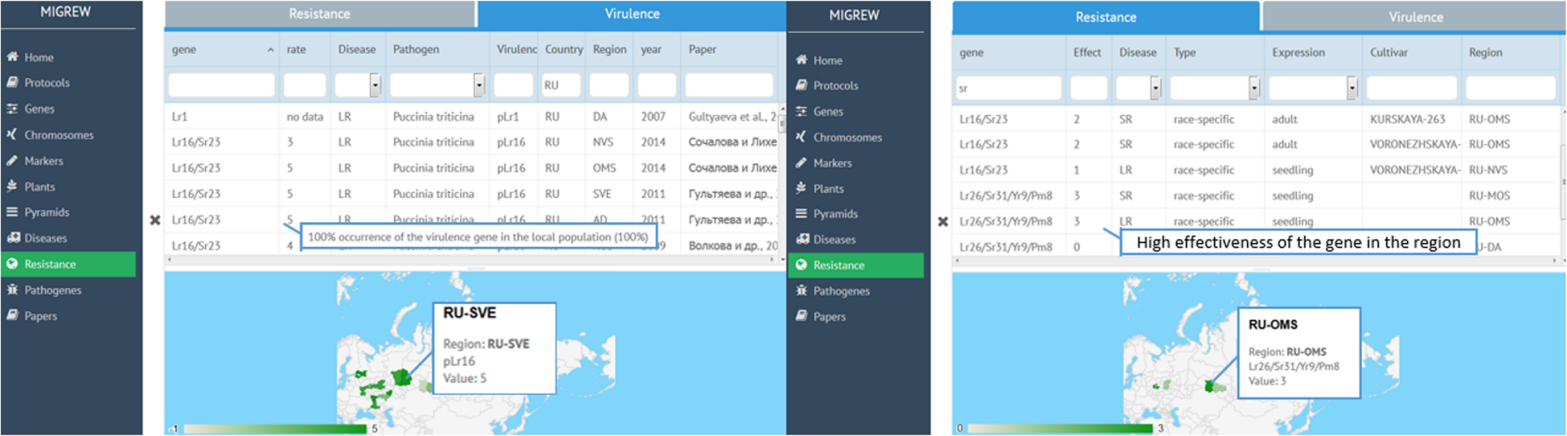
Virulence description page (left). Resistance description page (right)

After choosing the tab “Resistance” in the menu, one can go to the “Resistance” page or to the “Virulence” page, both according with search bar and interactive map (Fig. 9).

The “Resistance” page provides data on effectiveness of the wheat resistance gene or group of co-segregated resistance genes in the regions of Russia. The “Virulence” page allows one to get information about occurrence rate of the virulence gene in the pathogen populations of Russia. Maps show distributions of wheat resistance genes or virulence genes of pathogens. Regions on map are given according to the ISO_3166–2 standard. The region is highlighted and a tooltip is appeared only in case MIGREW contains appropriate data on resistance or virulence. The user is able to update information through the Feedback option. Pathogens information is available in additional menu tab “Pathogens”. It shows the list of pathogens stored in the database with search bar for virulence genes symbols.

To search information from particular paper, one can use the “Papers” menu tab. It contains the list of publications stored in the database with the set of filters that help to find the paper(s) of interest. On the paper description page, one can find data associated with the selected paper such as Genes, Markers and Protocols.

## Discussion

In this paper, we present a database with user-friendly interface that will be of interest for wheat researchers and breeders. We also present several examples to guide plant pathologists and breeders using the conventional and molecular methods.

Defense against pathogens is an important problems of modern agriculture. Plant disease resistance genes detect pathogen attacks and facilitate a counter strike against pathogens [3–6]. The information on avirulent genes in migrating fungal populations together with the resistant genes and translocations in wheat lines is important to be incorporated into breeding programs. The user (for example, a wheat pathologist) can obtain virulence distribution in pathogen populations using the MIGREW. Clicking the tab “Resistance and virulence genes” in the Homepage, the user will see the map of the Russian Federation. Among the regions there are active tabs where monitoring survey of wheat was carried out by the leading Russian research institutes: All-Russian Research Institute of Phytopathology, All-Russian Institute for Plant Protection, All-Russian Biological Plant Protection Research Institute, Agricultural Research Institute for South-East Regions of Russia, Samara Agricultural Research Institute, Сhelyabinsk Research Institute of Agriculture, Omsk State Agricultural University, Siberian Research Institute of Plant Industry and Breeding, Institute of Cytology and Genetics, etc.

Compared with other information resources, like “Agro Ecological Atlas of Russia and Neighboring Countries” (http://www.agroatlas.ru), the MIGREW database has a more intuitive graphical user interface and an option to download new relevant data.

If the user is a plant breeder using conventional methods, he/she can obtain the data on effectiveness of wheat resistance genes exploited in local cultivars and varieties including their effectiveness to the corresponding fungal pathogen from the foliar disease complex of wheat and its dynamics.

Selecting the “Diseases” tab in the Homepage, the user will see the pictures of foliar diseases of wheat and descriptions. To obtain the list of resistance genes against disease under interest, the user has to select the tab. Every gene symbol is linked with data on effectiveness against diseases; molecular markers for genotyping; pyramids including this gene; plants where this gene was identified with links to corresponded publications. The name of varieties are available both in English (as they indicated in the GRIS database) and in Russian for convenient search of an object in the database.

If the user is a breeder operating MAS techniques, it is possible to get information about chromosome localization of genes, molecular markers and verifying protocols. Selecting the “Chromosomes” tab in the Homepage, the user can find the localization of a gene under interest on wheat chromosomes with a search bar. All tabs are clickable here: there are links between genes and appropriate markers.

Selecting the “Protocols” of molecular markers tab in the Homepage, the user will see a common list of markers for each resistance gene. The tab “marker” contains general information on molecular markers of resistance genes, most often mentioned in experimental articles on the immunity of cereals. It also indicates the ability of the marker to identify the dominant allele of the gene or the dominant and recessive alleles (codominant markers). Among other things, the molecular technique for obtaining the marker and the nucleotide sequences of the direct (F) and reverse (R) primers used in the PCR amplification of molecular markers are given. The window contains the active tab “Marker visualization”. Clicking on it, the user will see information about setting the PCR analysis, conducting electrophoresis.

## Conclusions

The MIGREW database has been developed as a unified web-based interface the information on fungi-wheat objects keeping the data available for users with different requests, breeders and plant pathologists.

MIGREW database currently contains data on (1) wheat varieties and breeding lines involved in breeding for immunity; (2) wheat resistance genes, their chromosome localization and molecular markers; (3) molecular marker protocols; (4) effectiveness of rust disease resistance genes in different regions of Russia; (5) virulence genes of fungi from foliar pathogen complex of wheat. Public access to the MIGREW is possible in two ways: via WEB application as the main user interface (migrew.sysbio.cytogen.ru, migrew.sysbio.cytogen.ru/migrew); via direct data access through the REST API (migrew.sysbio.cytogen.ru/migrew_api). The REST API makes real integration into bioinformatics pipelines. We are still working on the MIGREW database extending data accumulation and application experience areas. If a reader have any idea of the MIGREW improving, the authors would appreciate him/her to contact us.

As a brief overview of informational resources concerning the genetics of wheat immunity to fungal disease, there is a comparison of the MIGREW database with two Russian databases, the GRIS and the Agroecological Atlas, and with the American resource MASwheat. The MASwheat database served the marker assisted selection of disease resistance, quality and agronomic traits. In addition to molecular markers protocols, the MIGREW database provides an extensive list of wheat cultivars and varieties where specific resistance genes were identified. At present, there is primarily Russian germplasm in the MIGREW database partially extracted from the GRIS database. The MIGREW also accumulates the data on virulence genes of foliar fungal disease of wheat, which are partly shared with the resource “Agro Ecological Atlas of Russia and Neighboring Countries” (http://www.agroatlas.ru). The MIGREW database is distinguished by different technologies to access and display the information with more intuitive interface and an option to download new relevant data.

Most publications on Russian germplasm are presented in local journals that are inaccessible to scientists abroad. It is assumed that the database partially compensates for the lack of data in the foreign press on the genetics and originators of Russian breeding lines and wheat varieties, as well as on the virulence of the population structure of pathogens of the Russian population affecting the wheat crop.

## Additional file 1

Additional file 1: Python script that demonstrates the MIGREW database API usage. (PDF 98 kb)
