# Supplementary material for: MIGREW: database on molecular identification of genes for resistance in wheat
Source: BMC Bioinformatics. 2019 Feb 5;20(Suppl 1):36. doi: 10.1186/s12859-018-2569-4 (PMC6362583; doi:10.1186/s12859-018-2569-4)
Supplement: Supplementary file 1 — Python script that demonstrates the MIGREW database API usage. (PDF 98 kb) [file 12859_2018_2569_MOESM1_ESM.pdf]

```
import requests
import json

PathToAPI='http://migrew.sysbio.cytogen.ru/migrew_api'

#%% Get list of Diseases
url = PathToAPI + '/diseases'
resp=requests.get(url)
diseases= resp.json()
for dis in diseases:
    print('ID: ' + dis['identifier']+'; name: ' + dis['name']+'; in russian: '+dis['name_rus']+'; description: ‘ +
dis['description'])

#%% Get list of genes associated with the seclected Disease
diseaseId = 'SR'  # Stem Rust

RequestURL = PathToAPI+'/diseases/'+diseaseId +'/genes'
resp=requests.get(RequestURL)                                    # run a request.
genesList = resp.json()
for gene in genesList:
    print(
    'ID:'+str(gene['dbid'])+';'
    +' Name:'+gene['identifier']+';'
    +' Chromosome:'+gene['chromosome']
    )

#%% Get list of Markers
url = PathToAPI + '/markers'
resp=requests.get(url)
markers= resp.json()
for marker in markers:
    print('Name: ' + marker['identifier']+'; InheritanceType: ' + marker['inheritanceType'] )

#%% Get the list of Markers associated genes
markerName = 'pwm16'
url = PathToAPI + '/markers/'+markerName +'/genes'
resp=requests.get(url)
genes= resp.json()
print('With marker '+markerName+' we have the next associated genes:' )
for gene in genes:
    print( 'gene_id: '+gene['gene_id']+ ';'
         +' gene: ' + gene['gene']+';'
         +' distance: ' + ('no data' if gene['distance']<0 else gene['distance']) + ';'
         +' paper: '+gene['paper'] )
```
